# The BET inhibitor sensitivity is associated with the expression level of CDC25B in pancreatic cancer models

**DOI:** 10.20517/cdr.2024.53

**Published:** 2024-10-18

**Authors:** Aubrey L. Miller, Patrick L. Garcia, Rebecca B. Vance, Eric O. Heard, Eric J. Brown, Karina J. Yoon

**Affiliations:** Department of Pharmacology and Toxicology, Heersink School of Medicine, University of Alabama at Birmingham, Birmingham, AL 35294, USA.

**Keywords:** Cell division cycle 25B (CDC25B), pancreatic ductal adenocarcinoma (PDAC), gemcitabine, patient-derived xenograft (PDX) models, BET inhibitor (BETi), gemcitabine-resistant (gemR) models

## Abstract

**Aim:** Cell division cycle 25B (CDC25B) belongs to the CDC25 family of phosphatases that regulate cell cycle progression. CDC25B also contributes to tumor initiation and progression, but no connection between CDC25B levels and drug sensitivity in pancreatic cancer has been reported. Based on our finding that bromodomain and extraterminal domain (BET) inhibitors decrease levels of CDC25B, we aim to compare the sensitivity of models expressing contrasting levels of CDC25B to the BET inhibitor JQ1, in pancreatic cancer cell lines *in vitro* and in patient-derived xenograft (PDX) models of pancreatic ductal adenocarcinoma (PDAC) *in vivo*.

**Methods:** We compared the efficacy of the standard of care agent gemcitabine with the BET inhibitor JQ1, using alamarBlue assays to determine IC_50_s of three pancreatic cancer cell lines *in vitro*. We used immunohistochemistry (IHC) and immunoblot (IB) to detect CDC25B. We also compared the effect of each agent on the progression of PDX models of PDAC *in vivo* with contrasting levels of CDC25B.

**Results:** Immunohistochemical data demonstrated that levels of CDC25B differed by ~2- to 5-fold in cell lines and PDX models used. *In vitro* data showed that the level of CDC25B paralleled sensitivity to JQ1. Similarly, *in vivo* data showed that tumors with high-level CDC25B were more sensitive to JQ1 than tumors with lower CDC25B. The combination of JQ1 + a pan CDC25 inhibitor was synergistic in gemcitabine-resistant Panc1.gemR cells that had relatively high levels of CDC25B expression compared to parent cells.

**Conclusion:** The data suggest that CDC25B may be an independent indicator of sensitivity to BET inhibitors and that CDC25B may contribute to gemcitabine insensitivity in this tumor type.

## INTRODUCTION

Pancreatic ductal adenocarcinoma (PDAC), the most common type of pancreatic cancer, is estimated to account for ~52,000 deaths in the US in 2024^[1]^. At diagnosis, 85% of PDAC patients present with locally advanced or metastatic disease^[2]^. These patients are ineligible for surgical resection, the only potentially curative treatment, and are treated most frequently with FOLFIRINOX or with gemcitabine-based regimens^[3,4]^. No biomarkers that predict response to either regimen have been reported.

Multiple studies have focused on identifying effective therapeutic targets for treating patients with pancreatic cancer. Recent efforts include identifying ways to alter the function of subtypes of cancer-associated fibroblasts (CAFs) known to support pancreatic tumor progression. For example, the Smoothened antagonist LDE225 inhibits the Hedgehog (HH) pathway, and this inhibition decreases the ratio of myofibroblasts (myCAFs) to inflammatory CAFs (iCAFs) and delays PDAC tumor growth in preclinical models^[5]^. Other efforts are based on the rationale that the DNA damage induced by gemcitabine, a PDAC standard of care, results in G2/M checkpoint activation and that targeting cell cycle checkpoints such as Chk1, Wee1, and ATR will augment the anti-tumor effect of gemcitabine. Most studies investigating the efficacy of checkpoint inhibitors have been evaluated in combination with gemcitabine. Preclinical and clinical studies include the use of gemcitabine in combination with Chk1 inhibitors AZD7762, LY2880070, and LY2603618^[6-9]^, the Wee1 inhibitor adavosertib (AZD1775)^[10]^, and the ATR inhibitor ceralasertib (AZD6738)^[11,12]^. Although these and other approaches^[13-15]^ have shown some efficacy in preclinical studies, additional data from clinical trials are needed to verify preclinical efficacy and develop effective personalized treatment strategies.

Cell division cycle 25B (CDC25B) is one of three CDC25 isoforms (CDC25A, B, C) responsible for regulating the G2/M transition by a mechanism involving dephosphorylation and activation of cyclin-dependent kinase (CDK) and cyclin complexes^[16-18]^. CDC25B was first designated as an oncogene when it was found to induce oncogenic transformation of mouse embryonic fibroblasts *in vitro* in cooperation with activated mutant H-Ras and to induce tumor formation in nude mice^[19]^. Overexpression of CDC25B has since been shown to abrogate the DNA damage checkpoint response and to induce premature mitosis. CDC25B is overexpressed in various types of human tumors, including PDAC^[20-24]^. The work of Guo *et al.* suggests that CDC25B represents a potential therapeutic target in PDAC^[20]^, but no CDC25- or CDC25B-specific inhibitors have been evaluated clinically.

Multiple studies associate CDC25B levels with specific clinicopathological parameters. In glioblastoma, increased CDC25B expression was associated with tumors of higher histological grade (*P* < 0.0001) and with shorter disease-free survival (*P* < 0.0001)^[25]^. Similarly, in non-small cell lung cancer, high levels of CDC25B correlated with angiogenic markers endothelin (*P* = 0.0002) and microvessels (*P* = 0.03) and with shorter overall and disease-free survival (*P* = 0.04)^[26]^. Interestingly, overexpression of CDC25B is associated with sensitivity to radiation in human esophageal cancers^[27]^.

Our lab reported that the bromodomain and extraterminal domain inhibitor (BETi) JQ1 had efficacy in 5 PDAC patient-derived xenograft (PDX) models evaluated^[28]^. JQ1 decreased CDC25B transcriptome in all 5 models while CDC25B protein levels in four JQ1-sensitive models by 1.5- to 2.3-fold. In contrast to published reports suggesting that the efficacy of JQ1 was due to decreased levels of c-MYC, JQ1 did not decrease c-MYC protein levels in our *in vivo* models. The goal of the current study was to determine whether the level of CDC25B in PDX tumor models is associated with sensitivity to BETi or to gemcitabine.

## METHODS

### Cell lines, antibodies, agents

Pancreatic cancer cell lines, Panc1, MIA PaCa-2, and BxPC3, were purchased from the American Type Culture Collection (Manassas, VA, USA). These cell lines were cultured in DMEM supplemented with 10% FBS and 2 mM L-glutamine. Primary antibodies used were: CDC25B (ab70927, abcam, Cambridge, MA, USA), α-tubulin (#2125, Cell Signaling, Danvers, MA, USA). Gemcitabine hydrochloride (G-4177, LC laboratories, Woburn, MA, USA) was dissolved in sterile phosphate-buffered saline solution for *in vitro* experiments, NSC 95397 (a pan-CDC25 inhibitor, Tocris Bioscience, Minneapolis, MN, USA) was dissolved in DMSO, and JQ1 (HY-13030, MedChemExpress, Monmouth Junction, NJ, USA) was dissolved in DMSO for *in vitro* experiments. The final concentration of DMSO was < 0.01% *in vitro*.

### *In vivo* PDX study

Animal studies were approved by the University of Alabama at Birmingham Institutional Animal Care and Use Committee (IACUC) and were performed in accordance with Animal Protocol Number (APN): 09186.

Four-week-old female SCID CB 17-/- mice were purchased from Taconic Farms (Newton, MA, USA) or Charles River (Wilmington, MA, USA) and housed in the AAALAC accredited vivarium, UAB Research Support Building and provided with food and water ad libitum. Tumors were implanted subcutaneously and bilaterally into mice. PDAC PDX models UAB-PA16 (PA16 CDC25B-low) or UAB-PA18 (PA18 CDC25B- high) were used. *N* = 7-9 tumors per group for both PA16 and PA18 studies (*N* = 8 for control and JQ1 and *N* = 7 for gemcitabine-treated PA16 tumors; *N* = 7 for control, *N* = 9 tumors for JQ1 and *N* = 8 gemcitabine-treated PA18 tumors). When tumors reached > 200 mm^3^, mice received intraperitoneal (ip) injections of either 100 mg/kg gemcitabine weekly or the vehicle control (saline) weekly for 5 weeks. The JQ1 cohorts received ip injections of 50 mg/kg JQ1 daily or control (10% DMSO in 10% beta-cyclodextrin, Sigma-Aldrich) daily for 21 days. The data for PA16 JQ1 cohorts were previously published^[29]^. Tumor size was measured twice a week using digital calipers and tumor volume was calculated using the formula v = (π/6)d^3^. Tumors were harvested 24 h after the final treatment and formalin-fixed and paraffin-embedded (FFPE) for future analysis. Tumor volumes are presented as mean ± SEM and compared using two-way analysis of variance (ANOVA) followed by Sidak multiple comparison post test (Prism).

### Immunohistochemistry

Immunohistochemical staining and analyses were performed as previously described^[28-31]^. Briefly, slides containing FFPE tumor sections were incubated overnight with primary antibody for CDC25B. The following day, the slides were stained with secondary antibody and DAB Chromagen and finally counterstained with hematoxylin. Images were taken on an Olympus BH-2 microscope with DP71 camera and DPA-BSW v3.1 software or a Zeiss Axio Observer Z.1 microscope with Zen 2 Blue imaging software (Zeiss). Expression Indices for CDC25B were calculated by assigning a staining intensity (0, 1, 2, 3) to a given percent of tumor cells in a field. The percentage was multiplied by the staining intensity and then these values were totaled to give a score between 0 and 300.

### Hematoxylin and eosin staining

Histological analysis was performed as previously described^[28-31]^. Images were taken on an Olympus BH-2 microscope with DP71 camera and DPA-BSW v3.1 software.

### Immunoblot

Whole-cell lysates were prepared in NP-40 lysis buffer with protease inhibitor cocktail and analysis was performed as previously described^[28,29,31,32]^. Briefly, 40 μg of protein was loaded on 10% Tris-Glycine gels and ran at 200 volts and semidry transferred to PVDF membranes. Proteins were then assessed using primary antibodies CDC25B and α-tubulin. Blots were quantitated using Image J (Image J, U.S. National Institutes of Health, Bethesda, Maryland, USA).

### Cell viability

Alamarblue cell viability assays were performed as previously described^[29,31,32]^. Briefly, cells were plated on 96-well tissue culture-treated plates. Serial dilutions of JQ1 were added to culture media for 72 h, or JQ1, CDC25 inhibitor (CDC25i), JQ1 + CDC25i, and JQ1 + gemcitabine for 96 h. AlamarBlue reagent was added to the wells according to manufacturer’s instructions and fluorescence was read on a Victor X5 microplate reader at 590 nm. Results are presented as the average ± SEM of three independent experiments.

### Statistics

All statistical analyses were performed using GraphPad Prism version 10. Statistical significance was calculated using one-way or two-way ANOVA unless otherwise specified. A *P* < 0.05 was considered significant.

## RESULTS

### A panel of PDAC PDX models express different levels of CDC25B

We reported previously that the bromodomain and extraterminal domain inhibitor (BETi) JQ1 had efficacy in five PDAC PDX models^[28]^. We observed that CDC25B was one of the transcripts most downregulated by JQ1 in all PDX models evaluated. Immunohistochemistry (IHC) and immunoblot (IB) of tumors exposed to JQ1 *in vivo* demonstrated that JQ1 decreased CDC25B protein levels by 1.5- to 2.3-fold^[28]^. The goal of the current study was to assess whether CDC25B is an independent indicator of sensitivity to BETi. We also wanted to determine if CDC25B levels reflect gemcitabine sensitivity since gemcitabine-based combination chemotherapy is a current standard of care for pancreatic cancer, and we have reported that a BETi + gemcitabine is synergistic *in vitro* in parent pancreatic cancer cell lines^[29]^.

**Figure 1:**
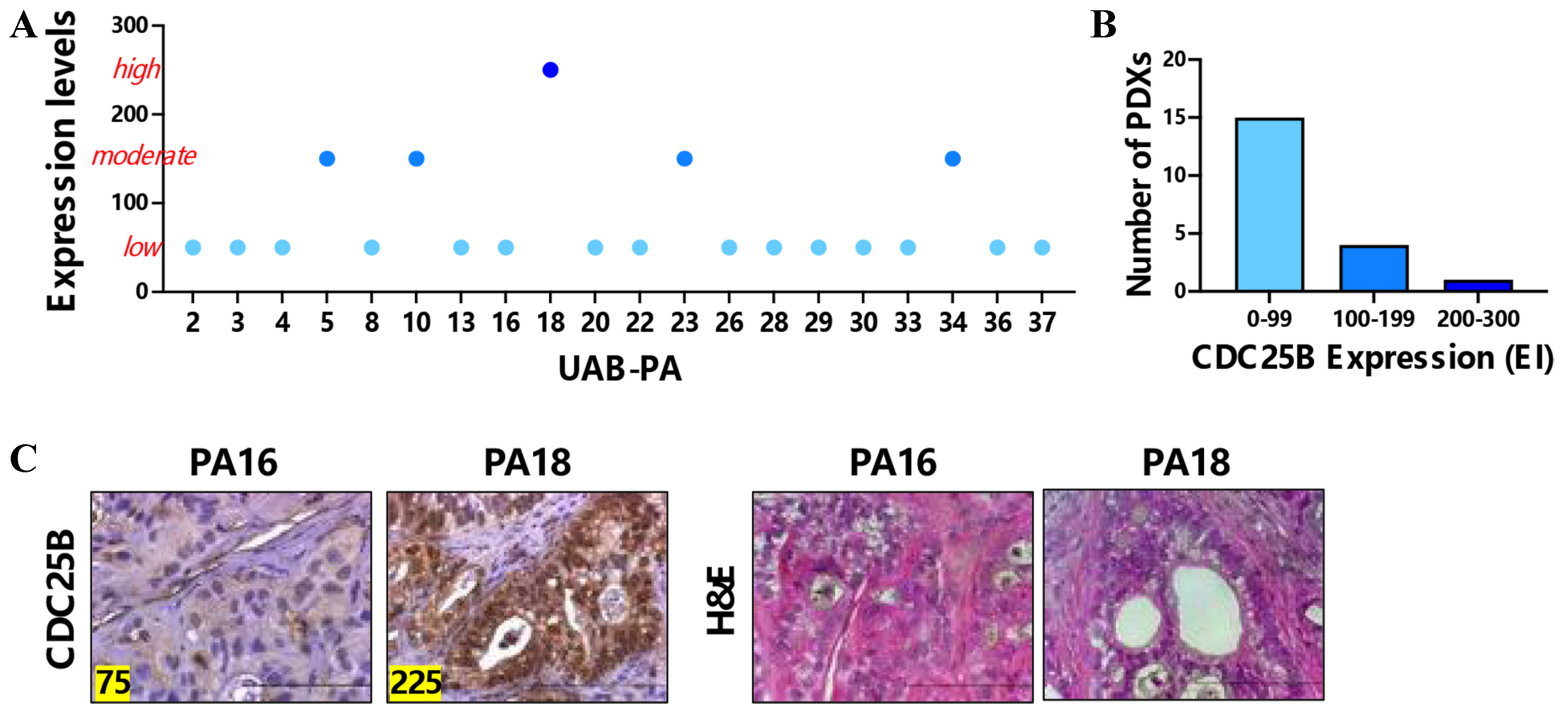
PDX models of PDAC have contrasting levels of CDC25B protein. (A) IHC of 20 PDAC PDX models shows that the models express different levels of CDC25B; (B) Bar graph depicting the number of tumors with low, moderate or high level CDC25B; (C) IHC staining for CDC25B in PA16 or PA18 PDAC PDX tumor models. Expression indices are shown in the left bottom corner of each image. The tumor sections were counterstained with H&E. Bar = 10 µm. PDX: Patient-derived xenograft; PDAC: pancreatic ductal adenocarcinoma; CDC25B: cell division cycle 25B; IHC: immunohistochemistry; H&E: hematoxylin and eosin.

We first evaluated protein levels of CDC25B in 20 of our panel of PDX models of PDAC, using IHC and FFPE sections. Expression was designated low, moderate, or high based on expression indices (low = EI 0-99, moderate = EI 100-199, and high = EI 200-300) [Figure 1A]. Our screen showed that 15/20 tumor models expressed low levels of CDC25B, 4/20 models expressed moderate levels, and 1/20 tumor models expressed high levels of CDC25B [Figure 1B]. We then used the PA16 CDC25B-low and the PA18 CDC25B-high PDX models to determine if the CDC25B level in these models of independent origin predicted sensitivity to BETi or to gemcitabine. Both of these models were derived from stage IIB, moderately to poorly differentiated tumors that expressed mutant KRAS G12D^[30]^. Figure 1C shows IHC staining for CDC25B in these tumor models. Hematoxylin and eosin (H&E) counterstain was used to assess general morphology.

### The BET inhibitor JQ1 suppressed the growth of PA18 CDC25B-high PDAC PDXs but had less efficacy for PA16 CDC25B-low tumors. Conversely, PA16 tumors responded better to gemcitabine than did PA18 tumors

**Figure 2:**
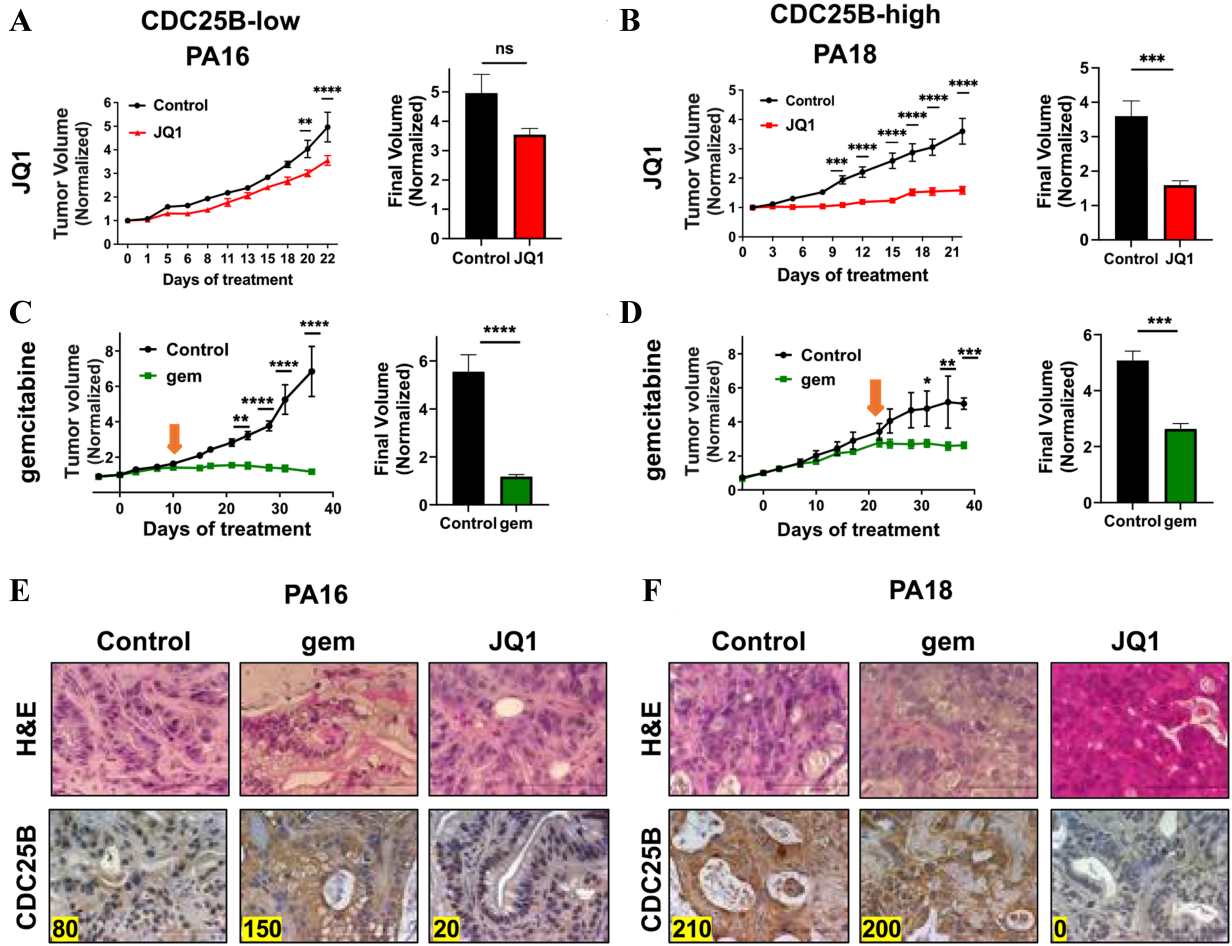
PA18 CDC25B-high tumors are sensitive to JQ1, but less sensitive to gemcitabine. JQ1 inhibits CDC25B expression. (A and B) Tumor growth inhibition in mice treated with JQ1 50 mg/kg daily for 3 weeks for PA16 (A) or PA18 (B) PDX tumors. Tumor volumes at the termination of treatment are shown in the right panels as bar graphs. *N* = 8 tumors/group for PA16; *N* = 7 for control and *N* = 9 tumors for JQ1 for PA18. Initial average tumor volumes (mm^3^) for PA16 were 174 (Control) and 214 (JQ1), and for PA18 were 546 (Control) and 446 (JQ1); (C and D) Tumor growth inhibition in mice treated with gemcitabine 100 mg/kg weekly for 5 weeks for PA16 (C) or P18 (D) PDX tumors. Final tumor volumes (tumor volumes on the last day of treatment) are compared in the right panels as bar graphs. Efficacy data were analyzed by two-way ANOVA followed by Sidak post test or unpaired *t* test (prism). ^*^P < 0.05, ^**^P < 0.01, ^***^P < 0.001, ^****^P < 0.0001. *N* = 8 for control and *N* = 7 for gemcitabine for PA16; *N* = 7 for control and *N* = 8 for gemcitabine for PA18. Initial average tumor volumes (mm^3^) for PA16 were 180 (Control) and 240 (gemcitabine), and for PA18 were 175 (Control) and 210 (gemcitabine); (E and F) Tumor tissue harvested from mice treated with control, gemcitabine, or JQ1 was stained with H&E and immunostained for CDC25B, for PA16 (E) or PA18 (F) tumors. Expression indices for CDC25B are shown in the left corner of each photomicrograph. CDC25B: Cell division cycle 25B; PDX: patient-derived xenograft; ANOVA: analysis of variance; H&E: hematoxylin and eosin.

We next evaluated the efficacy of the BETi JQ1 or gemcitabine in PA16 CDC25B-low and PA18 CDC25B-high PDX tumor models [Figure 2]. When tumors were > 200 mm^3^ in volume, mice bearing PA16 or PA18 PDX tumors were treated with JQ1 50 mg/kg ip daily for 3 weeks or gemcitabine 100 mg/kg weekly for 5 weeks. JQ1 as a single agent suppressed the growth of PA18 tumors for the duration of treatment (*P* < 0.0001), but only delayed PA16 tumor growth compared to controls [Figure 2A and B], Comparison of tumor volumes at completion of therapy (final tumor volume) showed a <1.5-fold difference between treated and control PA16 CDC25B-low tumors, and a >2.6-fold difference between treated and control PA18 CDC25B-high tumors (*P* < 0.001) [Figure 2A and B, right panels]. The data show that JQ1 was more effective in the CDC25B-high PDX model. During the treatment period, all mice maintained their body weight within 5% of pre-treatment weight [Supplementary Figure 1].

Efficacy data evaluating gemcitabine contrasted with data for JQ1. Gemcitabine suppressed the growth of PA16 CDC25B-low tumors during treatment (*P* < 0.0001) [Figure 2C]. In contrast, no response to gemcitabine was seen with PA18 CDC25B-high tumors until day 22 of treatment (orange arrow) [Figure 2D]. Comparison of tumor volumes at completion of therapy (final tumor volume) showed that gemcitabine inhibited the growth of PA16 tumors by ~7-fold and of PA18 tumors by <2-fold, compared to control [Figure 2C and D, right panels]. The difference in sensitivity cannot be attributed to the difference in tumor growth rate, since control PA16 and PA18 tumors grew at similar rates during the study period. Further, consistent with our previous publication^[28]^, IHC of FFPE sections of tumors exposed to drug showed that JQ1 decreased CDC25B expression in PA16 CDC25B-low tumors by 4-fold and in PA18 CDC25B-high tumors to EI = 0 [Figure 2E and F]. Of note, we observed that gemcitabine treatment increased CDC25B protein expression in PA16 CDC25B-low PDXs by ~2-fold [Figure 2E].

### Gemcitabine-resistant PDX tumors express higher levels of CDC25B than parent tumor counterparts

**Figure 3:**
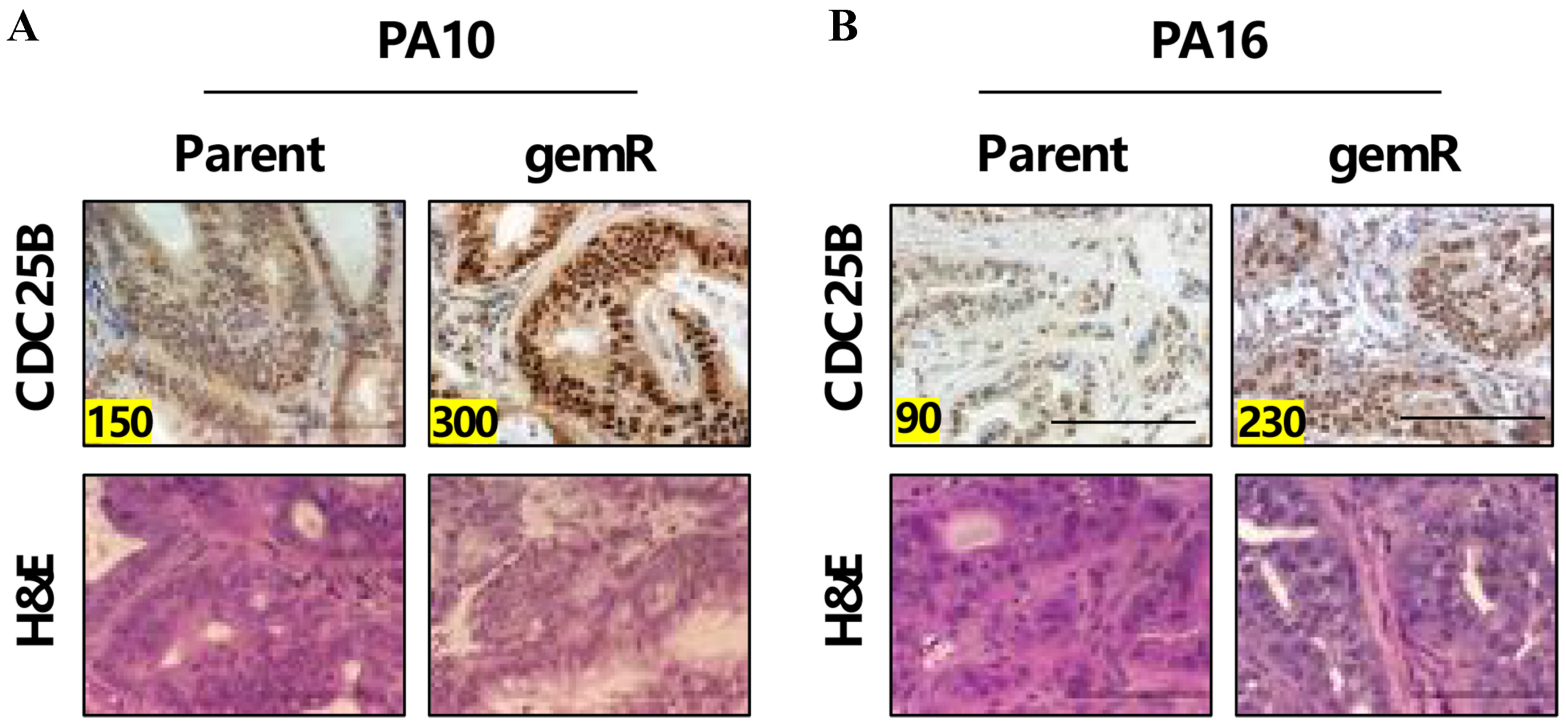
gemR PDX tumors have higher levels of CDC25B than parent tumors. (A) PA10- parent or gemR, or (B) PA16- parent or gemR PDX tumors were harvested, and sections were stained to detect CDC25B and counterstained with H&E to define nuclei. Bar = 10 µm. gemR: Gemcitabine-resistant; PDX: patient-derived xenograft; CDC25B: cell division cycle 25B; H&E: hematoxylin and eosin.

Because the literature documents an association between overexpression of CDC25B and drug and radiation resistance^[33,34]^, we next assessed whether gemcitabine-resistant (gemR) PDX models of PDAC had higher levels of CDC25B than the parent tumor counterparts. We previously developed PA10.gemR and PA16.gemR tumors by exposing parent tumors to gemcitabine *in vivo*, until gemcitabine was no longer growth-inhibitory^[35]^. Using these gemR PDX models, we stained with H&E and compared the levels of CDC25B in parent *vs.* gemR tumors, using IHC. We observed that gemcitabine treatment increased CDC25B by 2-fold at day 120 after initiation of gemcitabine 100 mg/kg treatment in PA10.gemR models [Figure 3A]. PA16.gemR tumors express 2.6-fold higher levels of CDC25B compared to their parent counterparts [Figure 3B].

### High CDC25B correlates with increased sensitivity to JQ1 *in vitro*

**Figure 4:**
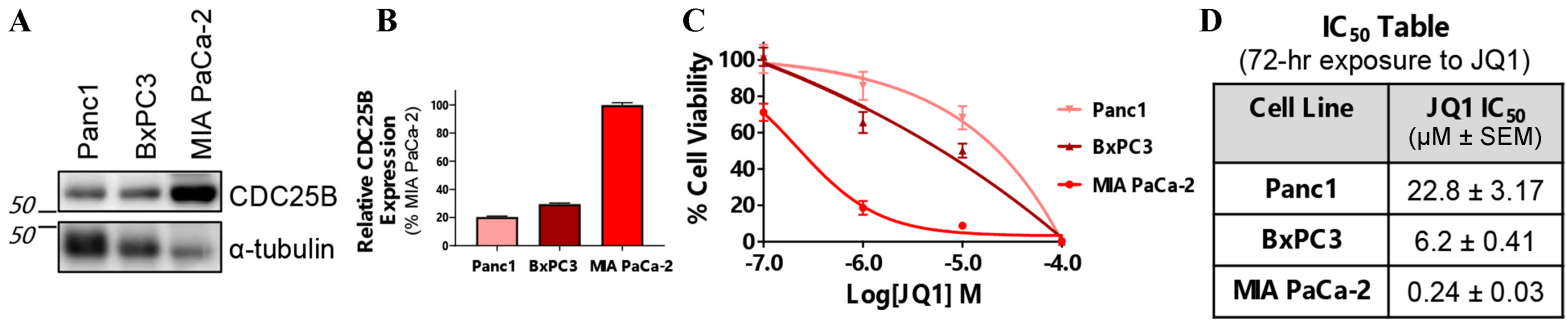
CDC25B-high pancreatic cancer cells are more sensitive to the BETi JQ1 than CDC25B-low cells. (A) IB showing the expression levels of CDC25B in Panc1, BxPC3, and MIA PaCa-2 pancreatic cancer cell lines; (B) A bar graph showing the quantitation of data in panel A. Quantitation was done using ImageJ. *N* = 3; (C) JQ1 sensitivity was assessed using alamarBlue cell viability assays after 72-hour exposure to JQ1. Data are presented as the mean ± SD. *N* = 3; (D) The table shows the IC_50_ values of each cell line. CDC25B: Cell division cycle 25B; BETi: bromodomain and extraterminal domain inhibitor; IB: immunoblot.

The observed difference in sensitivity to the BETi JQ1 in independently derived PA18 CDC25B-high PDX tumors compared to PA16 CDC25B-low tumors suggested that the level of CDC25B protein may be an independent indicator of sensitivity to BETi. To extend this observation, we evaluated CDC25B expression in three commonly used pancreatic cancer cell lines: Panc1, BxPC3, and MIA PaCa-2. IB analysis demonstrated that relative levels of CDC25B in these cell lines were MIA PaCa-2 > BxPC3 Panc1 cells [Figure 4A and B]. We then used alamarBlue assays to assess the effect of JQ1 (72-hour exposure) on cell viability [Figure 4C]. Cell sensitivity, as reflected by IC_50_, correlated with the level of CDC25B expression. MIA PaCa-2 was most sensitive to JQ1 (IC_50_ ~240 nM) compared to BxPC3 and Panc1 cells (IC_50_ ~6.2 and ~23 µM, respectively) [Figure 4D].

### JQ1 + CDC25 inhibitor and JQ1 + gemcitabine are synergistic in gemcitabine-resistant Panc1.gemR pancreatic cancer cells

**Figure 5:**
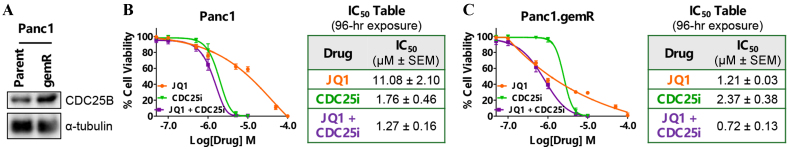
JQ1 + CDC25i decreases IC_50_s in Panc1 and gemcitabine-resistant Panc1.gemR cells. (A) IB data showing that Panc1.gemR cells express a higher level of CDC25B than parent Panc1 cells; (B) Sensitivity of Panc1 and (C) Panc1.gemR cells to JQ1 + CDC25i. Cells were exposed to JQ1, CDC25i, or JQ1 + CDC25i for 96 h and cell viability assessed using alamarBlue assays. IC_50_ values for JQ1, CDC25Bi, and the combination are shown in the right panel. *N* = 3. CDC25i: CDC25 inhibitor; gemR: gemcitabine-resistant; IB: immunoblot; CDC25B: cell division cycle 25B.

**Figure 6:**
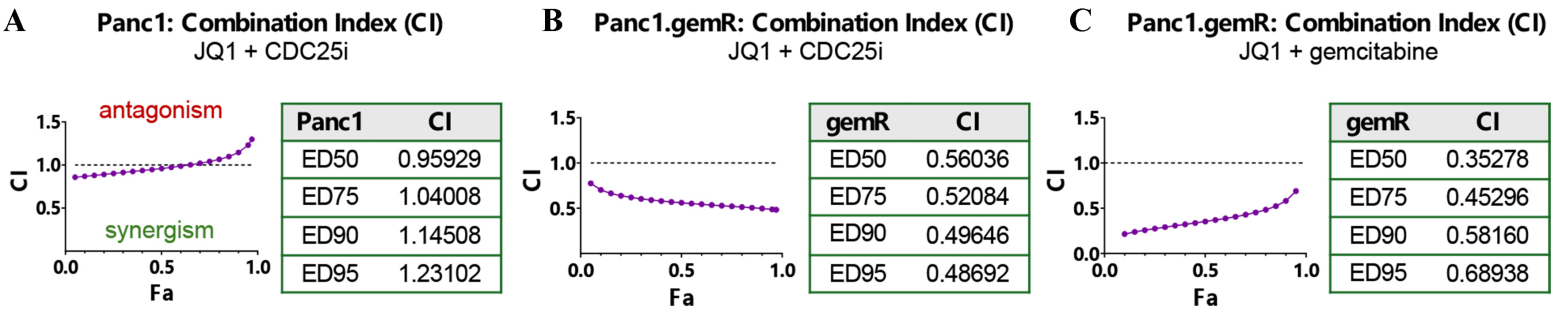
JQ1 + CDC25i or JQ1 + gemcitabine is synergistic in gemcitabine-resistant Panc1.gemR cells. (A and B) Fa-CI plot and CI table. CIs were calculated using compuSyn to determine whether JQ1 + CDC25i was antagonistic, additive, or synergistic. The CI < 0.5 indicates strong synergy; (C) Fa-CI plot and CI table to determine whether JQ1 + gemcitabine was synergistic in Panc1.gemR cells. The CI < 0.7 indicates synergy. CI = 1, < 1, or > 1 represents additive, synergistic, or antagonistic effects, respectively. CDC25i: CDC25 inhibitor; gemR: gemcitabine-resistant; Fa: Fraction affected; CI: combination index.

We reasoned that CDC25B would represent a potential drug target for PDAC, if the decrease in CDC25B contributed to the observed decrease in cell viability mediated by BETi JQ1. We postulated that JQ1-mediated inhibition of CDC25B expression plus inhibition of the activity of any residual CDC25B by a pan CDC25i would be synergistic. We tested this hypothesis by exposing Panc1 and Panc1.gemR cells to a pan CDC25 inhibitor, NSC 95397, and the BET inhibitor JQ1 *in vitro*. The Panc1.gemR cell line is ~3-logs less sensitive to gemcitabine than the parent Panc1 cell line^[36]^, and expresses a higher level of CDC25B than the parent Panc1 cell line [Figure 5A]. When exposed to JQ1 or CDC25i as single agents for 96 h, Panc1.gemR cells were ~9-fold more sensitive to JQ1 and ~1.3-fold less sensitive to CDC25i than parent Panc1 cells [Figure 5B and C]. However, Panc1.gemR cells were more sensitive to JQ1 + CDC25i combination by ~1.8-fold compared to Panc1 cells. Although the JQ1 + CDC25i was weakly antagonistic in Panc1 CDC25B-low cells [Figure 6A], this combination was synergistic in Panc1.gemR CDC25B-high cells at all concentrations evaluated (ED50, ED75, ED90, and ED95) [Figure 6B]. Further, we recently reported that JQ1 + gemcitabine was synergistic in parent pancreatic cancer cell lines *in vitro*^[29]^. We show here that gemcitabine-resistant Panc1.gemR cells express higher levels of CDC25B than Panc1 cells [Figure 5A] and that JQ1 + gemcitabine is synergistic in Panc1.gemR cells *in vitro* [Figure 6C]. The data suggest that CDC25B warrants further investigation as a potential drug target in overcoming gemcitabine resistance in this tumor type.

## DISCUSSION

This study characterizes levels of CDC25B expression in our series of 20 pancreatic PDX tumor models. We used two models and three pancreatic cancer cell lines with contrasting levels of CDC25B to compare the efficacy of the BET inhibitor JQ1, which inhibits the expression of CDC25B, or of gemcitabine, a standard of care for this tumor type. The data show that the CDC25B-high PDX tumor model was more sensitive to JQ1 and less sensitive to gemcitabine *in vivo* than the CDC25B-low model. Similar results were seen with cell lines *in vitro*. All models were of independent origin. We also observed that gemR PDX tumors express higher levels of CDC25B than their parent counterparts. AlamarBlue cell viability assays demonstrated that combinations of JQ1 + a pan CDC25 inhibitor and of JQ1 + gemcitabine were synergistic in a gemR pancreatic cancer cell line. These findings suggest that CDC25B level may be a marker of or contribute to BET inhibitor sensitivity in this tumor type, and that JQ1 sensitizes gemR cells to CDC25 inhibitors and to gemcitabine.

CDC25B belongs to the family of dual specific phosphatases that regulate entry into mitosis^[16,17,37]^. In the presence of DNA damage, CDC25B is under-phosphorylated, resulting in minimal nuclear localization to nuclei and leading to arrested cell cycle progression. Overexpression of CDC25B mimics phosphorylation of CDC25B and allows progression through the cell cycle, irrespective of phosphorylation status or DNA damage^[38]^. The overexpression of CDC25B we observed in gemR tumors is consistent with unregulated cell cycle progression seen with tumor progression. Other studies in the literature document CDC25B overexpression in multiple tumor types, including pancreatic, gastric, esophageal, and prostate cancers^[20,39-41]^. Further, this overexpression contributes to tumor initiation and progression and is associated with poor clinical outcomes^[41-45]^. Overexpression of CDC25B is also associated with drug resistance^[34,46,47]^. In contrast, overexpression of CDC25B in esophageal cancer models correlates with sensitivity to radiation^[27]^. Doxorubicin and PI3K-Akt-mTOR inhibitors increase CDC25B levels by transcriptional upregulation^[47]^. Similar to our finding that gemR cells have relatively high levels of CDC25B, upregulation of this protein by PI3K-Akt-mTOR inhibitors is associated with resistance to this family of inhibitors in AML^[46]^.

Efforts to develop anti-tumor agents that target CDC25B have been only marginally successful. Most of the first inhibitors were designed to inhibit the phosphatase activity of CDC25B by binding to the catalytic site^[48,49]^. More recent inhibitors are designed to disrupt CDC25B-CDK2/Cyclin A protein-protein interaction^[50,51]^ or to inhibit CDC25B expression by inhibiting TGFBR1-related signaling^[42]^.

Our finding that a BET inhibitor + CDC25 inhibitor is synergistic in gemR pancreatic cancer cells has not been reported previously. The molecular mechanism underlying this synergy is unknown. However, we have shown that JQ1 inhibits the expression of CDC25B, and CDC25i (NSC 95397) inhibits the activity of CDC25 proteins. The data suggest that combining JQ1 with NSC 95397 targets CDC25B by two distinct independent mechanisms: decreased expression and decreased activity. We propose that the CDC25i inhibits the activity of residual CDC25B, resulting in cell cycle stasis and inhibition of tumor progression. We have also shown that JQ1 + gemcitabine is synergistic in gemcitabine-resistant Panc1.gemR cells. Repeated exposure of PDAC cells *in vitro* or tumor models *in vivo* to gemcitabine increases levels of CDC25B. The resulting gemR cells or tumors express higher levels of CDC25B than their parent counterparts, and cells with high CDC25B expression are more sensitive to JQ1 than those with low CDC25B expression. We propose that the gemcitabine-mediated increase in CDC25B sensitizes the cells to JQ1 and that this increase is at least in part responsible for the synergy observed with JQ1 + CDC25i. Current work focuses on investigating the above hypotheses.

## DECLARATIONS

### Availability of data and materials

All datasets and materials generated in this study can be provided by the corresponding author upon reasonable request.

### Copyright

© The Author(s) 2024.

## References

[1] Siegel RL, Giaquinto AN, Jemal A (2024). Cancer statistics, 2024. CA Cancer J Clin.

[2] Liu M, Wei AC (2024). Advances in surgery and (Neo) adjuvant therapy in the management of pancreatic cancer. Hematol Oncol Clin North Am.

[4] Von Hoff DD, Ervin T, Arena FP (2013). Increased survival in pancreatic cancer with nab-paclitaxel plus gemcitabine. N Engl J Med.

[5] Steele NG, Biffi G, Kemp SB (2021). Inhibition of hedgehog signaling alters fibroblast composition in pancreatic cancer. Clin Cancer Res.

[6] Chung S, Vail P, Witkiewicz AK, Knudsen ES (2019). Coordinately targeting cell-cycle checkpoint functions in integrated models of pancreatic cancer. Clin Cancer Res.

[7] Huffman BM, Feng H, Parmar K (2023). A phase I expansion cohort study evaluating the safety and efficacy of the CHK1 inhibitor LY2880070 with low-dose gemcitabine in patients with metastatic pancreatic adenocarcinoma. Clin Cancer Res.

[8] Laquente B, Lopez-Martin J, Richards D (2017). A phase II study to evaluate LY2603618 in combination with gemcitabine in pancreatic cancer patients. BMC Cancer.

[9] Barnard D, Diaz HB, Burke T (2016). LY2603618, a selective CHK1 inhibitor, enhances the anti-tumor effect of gemcitabine in xenograft tumor models. Invest New Drugs.

[10] Cuneo KC, Morgan MA, Sahai V (2019). Dose escalation trial of the Wee1 inhibitor adavosertib (AZD1775) in combination with gemcitabine and radiation for patients with locally advanced pancreatic cancer. J Clin Oncol.

[11] Dunlop CR, Wallez Y, Johnson TI (2020). Complete loss of ATM function augments replication catastrophe induced by ATR inhibition and gemcitabine in pancreatic cancer models. Br J Cancer.

[12] Wallez Y, Dunlop CR, Johnson TI (2018). The ATR inhibitor AZD6738 synergizes with gemcitabine in vitro and in vivo to induce pancreatic ductal adenocarcinoma regression. Mol Cancer Ther.

[13] Schepis T, De Lucia SS, Pellegrino A (2023). State-of-the-art and upcoming innovations in pancreatic cancer care: a step forward to precision medicine. Cancers.

[14] Li Petri G, Pecoraro C, Randazzo O (2020). New imidazo[2,1-b][1,3,4]thiadiazole derivatives inhibit FAK phosphorylation and potentiate the antiproliferative effects of gemcitabine through modulation of the human equilibrative nucleoside transporter-1 in peritoneal mesothelioma. Anticancer Res.

[15] Pecoraro C, De Franco M, Carbone D (2023). 1,2,4-amino-triazine derivatives as pyruvate dehydrogenase kinase inhibitors: synthesis and pharmacological evaluation. Eur J Med Chem.

[16] Boutros R, Lobjois V, Ducommun B (2007). CDC25 phosphatases in cancer cells: key players?. Nat Rev Cancer.

[17] Lammer C, Wagerer S, Saffrich R, Mertens D, Ansorge W, Hoffmann I (1998). The cdc25B phosphatase is essential for the G2/M phase transition in human cells. J Cell Sci.

[18] Lindqvist A, Källström H, Lundgren A, Barsoum E, Rosenthal CK (2005). Cdc25B cooperates with Cdc25A to induce mitosis but has a unique role in activating cyclin B1-Cdk1 at the centrosome. J Cell Biol.

[19] Galaktionov K, Lee AK, Eckstein J (1995). CDC25 phosphatases as potential human oncogenes. Science.

[20] Guo J, Kleeff J, Li J (2004). Expression and functional significance of CDC25B in human pancreatic ductal adenocarcinoma. Oncogene.

[21] Li R, Wu B, Xia J, Ye L, Yang X (2019). Circular RNA hsa_circRNA_102958 promotes tumorigenesis of colorectal cancer via miR-585/CDC25B axis. Cancer Manag Res.

[22] Li S, Peng F, Ning Y (2020). SNHG16 as the miRNA let-7b-5p sponge facilitates the G2/M and epithelial-mesenchymal transition by regulating CDC25B and HMGA2 expression in hepatocellular carcinoma. J Cell Biochem.

[23] Liu JC, Granieri L, Shrestha M (2018). Identification of CDC25 as a common therapeutic target for triple-negative breast cancer. Cell Rep.

[24] Singh L, Pushker N, Sen S (2015). Expression of CDC25A and CDC25B phosphatase proteins in human retinoblastoma and its correlation with clinicopathological parameters. Br J Ophthalmol.

[25] Nakabayashi H, Hara M, Shimizu K (2006). Prognostic significance of CDC25B expression in gliomas. J Clin Pathol.

[26] Boldrini L, Gisfredi S, Ursino S, Lucchi M, Mussi A, Fontanini G (2007). CDC25B: relationship with angiogenesis and prognosis in non-small cell lung carcinoma. Hum Pathol.

[27] Miyata H, Doki Y, Shiozaki H (2000). CDC25B and p53 are independently implicated in radiation sensitivity for human esophageal cancers. Clin Cancer Res.

[28] Garcia PL, Miller AL, Kreitzburg KM (2016). The BET bromodomain inhibitor JQ1 suppresses growth of pancreatic ductal adenocarcinoma in patient-derived xenograft models. Oncogene.

[29] Miller AL, Garcia PL, Fehling SC (2021). The BET inhibitor JQ1 augments the antitumor efficacy of gemcitabine in preclinical models of pancreatic cancer. Cancers.

[30] Garcia PL, Council LN, Christein JD (2013). Development and histopathological characterization of tumorgraft models of pancreatic ductal adenocarcinoma. PLoS One.

[31] Miller AL, Fehling SC, Garcia PL (2019). The BET inhibitor JQ1 attenuates double-strand break repair and sensitizes models of pancreatic ductal adenocarcinoma to PARP inhibitors. EBioMedicine.

[32] Garcia PL, Miller AL, Zeng L, van Waardenburg RCAM, Yang ES, Yoon KJ (2022). The BET inhibitor JQ1 potentiates the anticlonogenic effect of radiation in pancreatic cancer cells. Front Oncol.

[33] Kang CM, Cho HN, Ahn JM (2004). Alteration of gene expression during radiation-induced resistance and tumorigenesis in NIH3T3 cells revealed by cDNA microarrays: involvement of MDM2 and CDC25B. Carcinogenesis.

[34] Pan X, Xu C, Cheng G, Chen Z, Liu M, Mei Y (2024). Transcription factor E2F3 activates CDC25B to regulate DNA damage and promote mitoxantrone resistance in stomach adenocarcinoma. Mol Biol Rep.

[35] Miller AL, Garcia PL, Gamblin TL, Vance RB, Yoon KJ (2020). Development of gemcitabine-resistant patient-derived xenograft models of pancreatic ductal adenocarcinoma. Cancer Drug Resist.

[36] Miller AL, Fehling SC, Vance RB (2024). BET inhibition decreases HMGCS2 and sensitizes resistant pancreatic tumors to gemcitabine. Cancer Lett.

[37] Thomas Y, Peter M, Mechali F, Blanchard JM, Coux O, Baldin V (2014). Kizuna is a novel mitotic substrate for CDC25B phosphatase. Cell Cycle.

[38] Kishi K, van Vugt MA, Okamoto K, Hayashi Y, Yaffe MB (2009). Functional dynamics of Polo-like kinase 1 at the centrosome. Mol Cell Biol.

[39] Bona AB, Calcagno DQ, Ribeiro HF (2020). Menadione reduces *CDC25B* expression and promotes tumor shrinkage in gastric cancer. Therap Adv Gastroenterol.

[40] Hu YC, Lam KY, Law S, Wong J, Srivastava G (2001). Identification of differentially expressed genes in esophageal squamous cell carcinoma (ESCC) by cDNA expression array: overexpression of Fra-1, Neogenin, Id-1, and CDC25B genes in ESCC. Clin Cancer Res.

[41] Ngan ES, Hashimoto Y, Ma ZQ, Tsai MJ, Tsai SY (2003). Overexpression of Cdc25B, an androgen receptor coactivator, in prostate cancer. Oncogene.

[42] Xiao Y, Yu Y, Gao D (2019). Inhibition of CDC25B with WG-391D impedes the tumorigenesis of ovarian cancer. Front Oncol.

[43] Ito Y, Yoshida H, Tomoda C (2005). Expression of cdc25B and cdc25A in medullary thyroid carcinoma: cdc25B expression level predicts a poor prognosis. Cancer Lett.

[44] Kudo Y, Yasui W, Ue T (1997). Overexpression of cyclin-dependent kinase-activating CDC25B phosphatase in human gastric carcinomas. Jpn J Cancer Res.

[45] Zhang Z, Zhang G, Kong C (2014). High expression of Cdc25B and low expression of 14-3-3σ is associated with the development and poor prognosis in urothelial carcinoma of bladder. Tumour Biol.

[46] Brenner AK, Reikvam H, Lavecchia A, Bruserud Ø (2014). Therapeutic targeting the cell division cycle 25 (CDC25) phosphatases in human acute myeloid leukemia--the possibility to target several kinases through inhibition of the various CDC25 isoforms. Molecules.

[47] Dalvai M, Mondesert O, Bugler B, Manenti S, Ducommun B, Dozier C (2013). Doxorubicin promotes transcriptional upregulation of Cdc25B in cancer cells by releasing Sp1 from the promoter. Oncogene.

[48] Brisson M, Nguyen T, Vogt A (2004). Discovery and characterization of novel small molecule inhibitors of human Cdc25B dual specificity phosphatase. Mol Pharmacol.

[49] George Rosenker KM, Paquette WD, Johnston PA (2015). Synthesis and biological evaluation of 3-aminoisoquinolin-1(2H)-one based inhibitors of the dual-specificity phosphatase Cdc25B. Bioorg Med Chem.

[50] Li HL, Ma Y, Ma Y (2017). The design of novel inhibitors for treating cancer by targeting CDC25B through disruption of CDC25B-CDK2/Cyclin A interaction using computational approaches. Oncotarget.

[51] Lund G, Dudkin S, Borkin D, Ni W, Grembecka J, Cierpicki T (2015). Inhibition of CDC25B phosphatase through disruption of protein-protein interaction. ACS Chem Biol.

