# Supplementary material for: The BET inhibitor sensitivity is associated with the expression level of CDC25B in pancreatic cancer models
Source: Cancer Drug Resist. 2024 Oct 18;7:40. doi: 10.20517/cdr.2024.53 (PMC11555179; doi:10.20517/cdr.2024.53)
Supplement: Supplementary file 1 [file cdr-7-40-SupplementaryMaterials.pdf]

Supplementary Materials

The BET inhibitor sensitivity is associated with the expression level of CDC25B in pancreatic cancer models

Aubrey L. Miller, Patrick L. Garcia, Rebecca B. Vance, Eric O. Heard, Eric J. Brown, Karina J. Yoon

[1]Department of Pharmacology and Toxicology, Heersink School of Medicine, University of Alabama at Birmingham, Birmingham, AL 35294, USA.

Correspondence to: Dr. Karina J. Yoon, Department of Pharmacology and Toxicology, Heersink School of Medicine, University of Alabama at Birmingham, 1670 University Blvd, Birmingham, AL 35294, USA. E-mail: kyoon@uab.edu

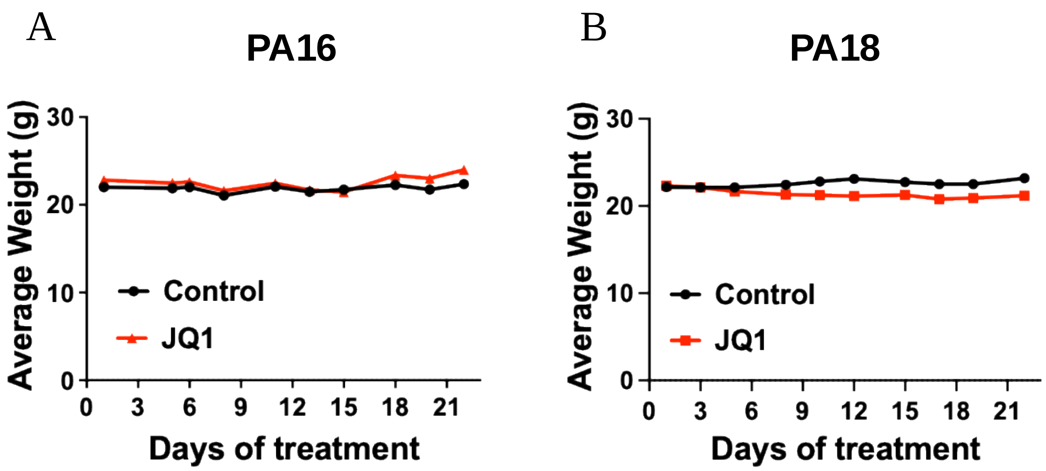

Supplementary Figure 1. Control and JQ1-treated mice bearing (A) PA16 or (B) PA18 PDX tumors maintained body weight. Mice were weighed every other day. These data are average weights for mice in experiments shown in Figures 2A and 2B, in the main text.
